# Modeling fomite‐mediated SARS‐CoV‐2 exposure through personal protective equipment doffing in a hospital environment

**DOI:** 10.1111/ina.12938

**Published:** 2021-10-24

**Authors:** Marco‐Felipe King, Amanda M. Wilson, Mark H. Weir, Martín López‐García, Jessica Proctor, Waseem Hiwar, Amirul Khan, Louise A. Fletcher, P. Andrew Sleigh, Ian Clifton, Stephanie J. Dancer, Mark Wilcox, Kelly A. Reynolds, Catherine J. Noakes

**Affiliations:** ^1^ School of Civil Engineering University of Leeds Leeds UK; ^2^ Department of Community, Environment, and Policy Mel and Enid Zuckerman College of Public Health University of Arizona Tucson Arizona USA; ^3^ Division of Environmental Health Sciences The Ohio State University Columbus Ohio USA; ^4^ School of Mathematics University of Leeds Leeds UK; ^5^ Department of Respiratory Medicine St. James's Hospital University of Leeds Leeds UK; ^6^ School of Applied Sciences Edinburgh Napier University Edinburgh UK; ^7^ Department of Microbiology Hairmyres Hospital NHS Lanarkshire Glasgow G75 8RG UK; ^8^ Healthcare Associated Infections Research Group Leeds Teaching Hospitals NHS Trust and University of Leeds Leeds UK

**Keywords:** COVID‐19, hospital infection model, PPE, quantitative microbial risk assessment, SARS CoV‐2, surface‐contact transmission

## Abstract

Self‐contamination during doffing of personal protective equipment (PPE) is a concern for healthcare workers (HCW) following SARS‐CoV‐2‐positive patient care. Staff may subconsciously become contaminated through improper glove removal; so, quantifying this exposure is critical for safe working procedures. HCW surface contact sequences on a respiratory ward were modeled using a discrete‐time Markov chain for: IV‐drip care, blood pressure monitoring, and doctors’ rounds. Accretion of viral RNA on gloves during care was modeled using a stochastic recurrence relation. In the simulation, the HCW then doffed PPE and contaminated themselves in a fraction of cases based on increasing caseload. A parametric study was conducted to analyze the effect of: (1a) increasing patient numbers on the ward, (1b) the proportion of COVID‐19 cases, (2) the length of a shift, and (3) the probability of touching contaminated PPE. The driving factors for the exposure were surface contamination and the number of surface contacts. The results simulate generally low viral exposures in most of the scenarios considered including on 100% COVID‐19 positive wards, although this is where the highest self‐inoculated dose is likely to occur with median 0.0305 viruses (95% CI =0–0.6 viruses). Dose correlates highly with surface contamination showing that this can be a determining factor for the exposure. The infection risk resulting from the exposure is challenging to estimate, as it will be influenced by the factors such as virus variant and vaccination rates.

**Practical Implications**

Infection risk from self‐contamination during doffing PPE is an important concern in healthcare settings, especially on a COVID‐19 ward. Fatigue during high workload shifts may result in an increased frequency of mistakes and hence the risk of exposure. Length of staff shift and a number of COVID‐19 patients on a ward correlate positively with the risk to staff through self‐contamination after doffing. Cleaning of far‐patient surfaces is equally important as cleaning traditional “high‐touch surfaces,” given that there is an additional risk from bioaerosol deposition outside the patient zone.

## 1 INTRODUCTION

Severe acute respiratory syndrome coronavirus 2 (SARS‐CoV‐2) is an enveloped virus which has infected in excess of 200 million people to date and caused more than four million deaths worldwide according to Johns Hopkins University's COVID‐19 dashboard.^1^ Inanimate objects known as fomites may host pathogens and have the potential to contribute to transmission in healthcare environments. This occurs in viral contamination spread^2^, ^3^, ^4^ including SARS‐CoV‐2.^5^, ^6^ However, it should be noted that there are uncertainties as to the relationship between the molecularly detected viruses and infectious viruses. In terms of persistence, there appears to be a similarity between SARS‐CoV‐1 and 2 on surfaces, where initial concentrations of 10^3.7^ median tissue culture infectious dose (TCID_50_)/ml (SARS‐CoV‐2) and of 10^3.4^ TCID_50_/ml (SARS‐1) reduced to 10^0.6^ TCID_50_/ml (SARS‐CoV‐2) and 10^0^.^7^ TCID_50_/ml (SARS‐1), respectively, due to the decay of viability of the virus after 72 h on plastic surfaces.^7^ Persistence on the scale of days under heavy contamination conditions allows an opportunity for exposure through hand‐to‐fomite contacts. Although personal protective equipment (PPE) such as gloves, gowns, and masks are worn to protect both patients and healthcare workers (HCW) from the exposure, self‐contamination during PPE doffing processes^8^, ^9^ poses risks to HCW and enables spread from one patient to another during multiple care episodes. SARS‐CoV‐2 has been detected on healthcare worker PPE^10^ and in the environment of rooms where doffing occurs, demonstrating that errors in doffing could facilitate COVID‐19 exposure and transmission.

While SARS‐CoV‐2 has been detected on PPE and patient surfaces, the relationship between viral RNA concentrations and risk of infection is still unknown.^11^ Bullard et al.^12^ present TCID_50_ and cycle threshold values relative to days since symptom onset, but these may not be translatable to concentrations on fomites due to the potential for more SARS‐CoV‐2‐genetic material corresponding to inactivated viruses resulting from incomplete surface disinfection practices. Quantitative microbial risk assessments (QMRA) involve the use of mathematical models to estimate doses of a pathogen and subsequent infection risk probabilities. Quantifying infection exposure and risk for any given dose can be used to guide intervention decision‐making and have been used in other public health contexts, such as in setting water quality standards.^13^ These typically rely on experimental doses of a microorganism inoculated into healthy participants or mice models in a known quantity. Whether they develop the infection can then be recorded.^13^ QMRA modeling and surface contact models have been used to evaluate multiple transmission pathways. The role of care‐specific behaviors in environmental microbial spread^14^ includes the effect of glove use in bacterial spread from one surface to another^15^ and evaluating risk reductions through hand hygiene or surface disinfection interventions.^16^, ^17^, ^18^ While a strength of QMRA is related to environmental monitoring data to health outcomes, a common limitation is the lack of specific human behavior data such as hand‐to‐face or hand‐to‐surface contact sequences that result in dose exposures.^18^, ^19^ The use of the QMRA modeling framework incorporating care type surface contact patterns before potential self‐contamination via PPE doffing will offer insight into viral exposure per shift.

The objective of this study is to relate SARS‐CoV‐2 concentrations on surfaces to predict the exposure of a single healthcare worker over an 8‐hour shift and estimate the effects of doffing mistakes and number of care episodes per shift on inoculated dose per shift.

## 2 METHODOLOGY

This approach combines human behavior and fomite‐mediated exposure models of 19 hospital scenarios, for which concentrations of SARS‐CoV‐2 on hands and infection risk for a single shift are estimated for a registered nurse, an auxiliary nurse, and a doctor. A control scenario was defined as a single episode of care with a SARS‐CoV‐2‐positive individual with an assumed 80% probability of self‐contamination during doffing: a “worst‐case scenario.” Eighteen other scenarios covered three likelihoods of self‐contamination: 10%, 50%, and 80%, ×2 caseload conditions: 7 patients (low) vs. 14 patients (high) × 3 probabilities of any given patient being COVID‐19 positive: low (5%), medium (50%), and a 100% COVID‐19‐positive ward. These rates of self‐contamination during doffing were assumed due to uncertainty as to how workload and stress, especially under pandemic conditions, would influence doffing. Exploring probabilities of self‐contamination as low as 5% and as high as 80% allows for the exploration of optimistic and worse‐case scenarios.

During low caseload conditions, it was assumed that the number of care episodes per shift would be less^7^ than that of the high load conditions.^14^ The assumed number of patient care episodes when PPE is worn per shift for low and high caseload scenarios were 7 and 14, respectively, based on a respiratory ward in a university teaching hospital in the UK. The low caseload estimate was based on the communication with a UK NHS consultant, who tracked the number of gowns used by healthcare workers over a week on a mixed COVID‐19 8‐bed respiratory ward. All model parameters are described and reported in Table 1. Per scenario, three simulations were run where sequences of hand‐surface contacts per care episode were care‐specific (IV care, observational care, or doctors’ rounds).

**TABLE 1:** Model parameters and their distributions/point values

Parameter	Distribution/point value	Reference
Surface contamination ($C_{\text{RNA}}$) (RNA/swabbed surface area)	For infected patient scenarios Surfaces: Triangular (min = 3.3 × 10^3^, mid=2.8 × 10^4^, max=6.6 × 10^4^) Patient: Point estimate: 3.3 × 10^3^	28
Area of any given surface ($A_{\text{surface}}$) (cm^2^)	Triangular (min = 5, max = 195, mid = 100)	Assumed
Fraction of RNA (infective) assumed to be infectious	Uniform (min = 0.001, max = 0.1)	Assumed
Finger‐to‐surface transfer efficiency (*β*) (fraction)	Normal (mean = 0.118, SD = 0.088) Left‐ and right‐truncated at 0 and 1, respectively	4
Surface‐to‐finger transfer efficiency (*λ*) (fraction)	Normal (mean = 0.123, SD = 0.068) Left‐ and right‐truncated at 0 and 1, respectively	4
Finger‐to‐mouth transfer efficiency (${\text{TE}}_{H®M}$) (fraction)	Normal (mean = 0.339, SD = 0.1318) Left‐ and right‐truncated at 0 and 1, respectively	50
Glove doffing self‐contamination transfer efficiency	Uniform (min = 3 × 10^−7^, max = 0.1)	8
*T*_99_ on Hands (h) used for calculating inactivation constants	Uniform (min = 1, max = 8)	24, 51
*T*_50_ on surfaces (h) used for calculating inactivation constants	Uniform (min = 4.59, max = 8.17)	7
Hand hygiene efficacy: alcohol gel (log_10_ reduction)	Uniform (min = 2, max = 4)	35
Hand hygiene efficacy: soap and water (log_10_ reduction)	Normal (mean = 1.62, SD = 0.12) Left‐and right‐truncated at 0 and 4, respectively	34
Fraction of total hand surface area for hand‐to‐mouth or hand‐to‐surface contacts ($S_{m}$ and $S_{h}$)	For in/out events: Uniform (min = 0.10, max = 0.17) For patient contacts: Uniform (min = 0.04, max = 0.25) For other surface contacts: Uniform (min = 0.008, max = 0.25) For hand‐to‐face contacts: Uniform (min = 0.008, max = 0.012)	26
Total hand surface area ($A_{h}$) (cm^2^)	Uniform (min = 445, max = 535)	19, 38
Dose response curve parameter^a^ *α*	0.36 ± 0.25 0.12, 19.6	46; this study
Dose response curve parameter^a^ *β*	5.94 ± 11.4 0.27, 802.1	46; this study

^a^ Dose response curve parameters are to be used in bootstrapped pairs. Mean ± SD and minimum and maximum are provided to offer context as to the magnitude of these parameters.

### 2.1 Healthcare worker surface contact behavior sequences

Fifty episodes of mock patient care were recorded overtly using videography in a respiratory ward side room at St James’ Hospital, Leeds. Mock care was undertaken by doctors and nurses with a volunteer from the research team to represent the patient. While these observations were carried out prior to COVID‐19, it is assumed that patient care would be similar for any infected patient, including a COVID‐19 patient. Ethical approval for the study was given by the NHS Health Research Authority Research Ethics Committee (London – Queen Square Research Ethics Committee), REF: 19/LO/0301. Sequences of surface contacts were recorded for three specific care types: IV drip insertion and subsequent care (IV, *n* = 17) conducted by registered nurses (RN); blood pressure, temperature, and oxygen saturation measurement (Observations, *n* = 20) conducted by auxiliary nurses; and doctors’ rounds (Rounds, *n* = 13). Data from care were used to generate representative contact patterns to model possible sequences of surface contacts by HCWs in a single patient room. Discrete Markov chains were used because HCWs were found to touch surfaces in a non‐random manner, insofar that transitional probabilities fit to observed behaviors from moving from one surface category were not all equal. By assigning each surface category a numerical value from 1 to 5, where *Equipment* = 1, *Patient* = 2, *Hygiene areas* = 3, *Near‐bed surfaces* = 4, and *Far‐bed surfaces* = 5, HCW sequential contact of surfaces can be modeled in terms of weighted probabilities.^14^ More information regarding the observation of these behaviors and analysis of sequences of events can be seen in King et al.^20^

The transition of an HCW between surface contacts is modeled using a discrete‐time Markov chain approach.^14^ Using defined weighted probabilities based on observation of patient care, surface contact by HCW can be simulated based on the property that given the present state, the future and past surfaces touched are independent. This is termed as the Markov property (Equation 1):

(1) $$P(X_{n+1}=i|X_{n}=j)$$

where $X_{n}$ represents the surface contacted in the *n*th event, *i* and *j* are two surfaces, and *P* represents a conditional probability. This is then denoted as $P_{j\to i}$ for the ease of notation. For example, the probability if the HCW is currently touching the table and they will next touch the chair is $P_{\text{table}\to \text{chair}}$ and can be worked out by counting the number of times this happens during care divided by the number of times any surface is touched after the table.^21^

Discrete‐time Markov chains were fitted to observed care contact sequences using the “Markov chain Fit” function from the R package *Markov chain (version 0*.*7*.*0)*. Separate Markov chains were fitted to IV care, doctors’ rounds, and observational care sequences. States included “in” (entrance to the patient room), “out” (exit from the patient room), contact with a far‐patient surface, contact with a near‐patient surface, contact with a hygiene surface (e.g., tap, sink, soap, or alcohol dispenser), and contact with equipment. For each episode of care, the first event was the entrance into the patient's room. It was assumed in the simulation that all HCWs wore a gown, gloves, mask, and face shield when entering the room in that hand‐to‐face contacts were not modeled during the episodes of care, and hand hygiene moments only occurred after doffing in between the care episodes. The episode of care ended when an “out” event occurred.

### 2.2 Exposure model

Accretion of microorganism on hands from the surface contacts has been demonstrated^14^ to respond to a recurrence relationship with the concentration on hands after the *n*th contact, $C_{n}^{h}$, with the concentration on hands, $C_{n‐1}^{h}$, and on the surface involved, $C_{n‐1}^{s}$, before the contact. See Equation (2).

(2) $$C_{n}^{h}=C_{n‐1}^{h}e^{‐k_{h}\Delta t}‐S_{h}\left(\lambda C_{n‐1}^{h}e^{‐k_{h}\Delta t}‐\beta C_{n‐1}^{s}e^{‐k_{s}\Delta t}\right)$$

This is an adaptation of the pathogen accretion model (PAM) from King et al.^14^ and a gradient transfer model by Julian et al.^22^ Here, the concentration on hands for contact *n* is equal to the previous concentration on the hand ($C_{n‐1}^{h}$) after adjusting for inactivation for the virus on the hand ($k_{h}$) and surface $k_{s}$, minus the removal from the hand due to the hand‐to‐surface transfer plus the gain to the hand due to surface‐to‐hand transfer. Δt is the time taken for an episode of patient care and sampled from a uniform distribution of range 2–20 min.^23^ Here, λ and β represent hand‐to‐surface and surface‐to‐hand transfer efficiencies, respectively. The fraction of the total hand surface area ($S_{h}$) is used to estimate how much virus is available for transfer, given a concentration of the number of viral particles/cm^2^ on the gloved hand and surface.

### 2.3 Estimating inactivation on the hand

Sizun et al. evaluated the survival of human coronaviruses (HCoV) strains OC43 and 229E on latex glove material after drying. Within 6 h, there was a reduction in viral infectivity for HCoV‐229E that we assume is equal to 99%.^24^ For HCV‐OC43, a reduction of approximately 99% in viral infectivity occurred within an hour.^24^ Harbourt et al.^25^ measured SARS CoV‐2 inactivation on pig skin with virus remaining viable for up to 8 h at 37°C. We, therefore, used a uniform distribution with a minimum of 1 h and a maximum of 8 h to estimate a distribution of $k_{h}$ inactivation rates.

### 2.4 Estimating inactivation on surfaces

The decay of the virus causing COVID‐19 has been shown to vary under both humidity and temperature, but in contrast with the previous findings,^7^ it appears that the surface material may not have a large impact on the decay rate.^25^ We, therefore, use one distribution of inactivation rates regardless of surface type by taking a conservative approach and using an averaged half‐life *τ* estimate for stainless steel‐ and plastic‐coated surfaces at 21–23°C^7^ at 40% relative humidity, which are 5.63 h (95%CI = 4.59–6.86 h) and 6.81 h (95%CI = 5.62–8.17 h), respectively. We assume a first‐order decay (Equation 3) to estimate the inactivation constant *k* which we use here for brevity instead of $k_{s}$ and $k_{h}$ in the Equation (2).

(3) $$C\left(t\right)=C_{0}e^{‐kt}$$

Surface viral concentration *C* at any given time t then depends uniquely on initial concentration $C_{0}$. Where the half‐life *τ*, is related to *k* by: $k_{s}=\log\left(2\right)/\tau$. Since the hospital rooms are made up of a combination of stainless steel and plastic surfaces, we have taken the widest confidence interval as bounds when sampling from a uniform distribution for inactivation rate $k_{s}$. Inactivation on gloves is assumed to be minimal for the time scale of a care episode (2–20 min).^23^

### 2.5 Fractional surface area

For contacts with the door handle during “in” or “out” behaviors, a fractional surface area was randomly sampled from a uniform distribution with a minimum of 0.10 and a maximum of 0.17 for open hand grip hand‐to‐object contacts.^26^ For contacts with the patient, a fractional surface area was randomly sampled from a uniform distribution with a minimum of 0.04 and a maximum of 0.25, for front partial finger or full front palm with finger contact configurations.^26^ For contacts with other surfaces, the fractional surface areas were randomly sampled from a uniform distribution with a minimum of 0.008 and a maximum of 0.25, spanning multiple contact and grip types from a single fingertip up to a full palm contact.^26^

### 2.6 Transfer efficiencies

All transfer efficiencies used in this model are unitless fractions ranging from 0 to 1, representing the fraction of viruses available for transfer that transfer from one surface to another upon contact. For contacts with surfaces other than the patient, a truncated normal distribution with a mean of 0.123 and a standard deviation of 0.068 with maximum 1 and minimum 0 was randomly sampled for surface‐to‐finger (*λ*) transfer efficiencies based on aggregated averages of influenza, rhinovirus, and norovirus.^4^ For patient contacts, transfer efficiencies were randomly sampled from a normal distribution with a mean of 0.056 and a standard deviation of 0.032, left‐ and right‐truncated at 0 and 1, respectively. The mean and standard deviation were informed by transfer efficiencies for rhinovirus measured for direct skin‐to‐skin contact.^27^ Transfer efficiencies from fingers to surfaces (*β*) are assumed to be normally distributed with a mean of 0.118 and a standard deviation of 0.088.^4^

### 2.7 Surface concentrations

If the patient was assumed to be infected, surface contamination levels (RNA/swab surface area) were sampled from a triangular distribution where the minimum and maximum were informed by minimum and maximum contamination levels reported for the surfaces in an intensive care unit ward.^28^ The median of these was used to inform the midpoint of the triangular distribution.^28^ For patient contacts, the concentration of virus detected on a patient mask was used as a point value (3.3 × 10^3^RNA/swab surface area).^28^ When a patient was not infected, it was assumed that contacts with surfaces and with the patient would not contribute to additional accretion of the virus on gloved hands.

Surface areas for relating concentrations of RNA/swabbed surface area reported by Guo et al. (2020) to RNA/cm^2^ were not provided. While a typical sampling size is 100 cm^2^, it may be as small as 10–25 cm^2^ ^29^, ^30^, ^31^, ^32^ and in the real‐world scenarios, sampling surface areas may be larger or smaller than these depending upon available surface area, ease of access, and the contamination magnitude expected. Since the surface areas of these surfaces were not provided, a triangular distribution (min = 5, max = 195, mid = 100) describing the surface area (cm^2^) of surfaces sampled was used to estimate RNA/cm^2^. Not all detected RNA was assumed to represent infectious viral particles. This is a conservative risk approach when utilizing molecular concentration data in QMRA.^33^ Therefore, concentrations on surfaces $C^{S}$ (viable viral particles/cm^2^) were estimated by Equation (4),

(4) $$C^{S}=\frac{C_{\text{RNA}}}{A_{\text{surface}}}\cdot \text{infective}$$

where $C_{\text{RNA}}$ is the RNA/swabbed surface area, $A_{\text{surface}}$ is the surface area (cm^2^) of the surface, and infective is the fraction of RNA that relates to infective viral particles (uniform(min = 0.001, max = 0.1)). This overlaps with a range used by Jones (2020) for COVID‐19 modeling. While data from Bullard et al. (2020) exist for relating molecularly detected SARS‐CoV‐2 to culturable SARS‐CoV‐2 for patient samples, these ratios do not translate to fomite scenarios where surface disinfection likely results in a more molecularly detectable viruses that do not translate to infectivity. Therefore, we did not use these data to inform our assumptions about viral infectivity for molecularly detected SARS‐CoV‐2 on surfaces.

### 2.8 Estimating exposure dose

For all scenarios, it was assumed that the starting concentration on gloved hands for the first episode of care was equal to 0 viral particles/cm^2^. If gloves were doffed and a new pair was donned in between care episodes, it was assumed that the next episode of care began with a concentration of 0 viral particles/cm^2^ on the gloved hands. After each care episode, a number was randomly sampled from a uniform distribution with a minimum of 0 and a maximum of 1. If this value was less than or equal to the set probability of self‐contamination during doffing, self‐contamination occurred, where the fraction of total virus was transferred from the outer glove surface to the hands was assumed to be uniformly distributed between 3 × 10–5% and 10%.^8^ There was then a 50/50 chance that either hand were washed or sanitized using alcohol gel due to the lack of available data describing proportions of hand hygiene attributable to these two methods occurring aftercare episodes. If they washed their hands, a log_10_ reduction was randomly sampled from a normal distribution with a mean of 1.62 and a standard deviation of 0.12 (min = 0 and max = 6).^34^ While these are not coronavirus‐specific handwashing efficacies they allow for a conservative estimate. If hand sanitizer was used, a log_10_ reduction was randomly sampled from a uniform distribution with a minimum of 2 and a maximum of 4.^35^

To estimate a dose, an expected concentration on the hands after doffing and hand hygiene was estimated, followed by an expected transfer to a facial mucosal membrane during a single hand‐to‐nose contact after each patient care episode (Equation 5).

(5) $$D=C_{h}\cdot {\text{TE}}_{\text{HM}}\cdot S_{m}\cdot A_{h}\cdot e^{‐k_{h}\Delta t}$$

There was a 50/50 chance that either the right or left hand was used for this hand‐to‐face contact, as contact patterns between right and left hands have been shown to lack statistically significant differences.^36^ Here, the transfer efficiency (*T*_H→M_) of the hand‐to‐nose contact was randomly sampled from a normal distribution with a mean of 33.90%, and a standard deviation of 13.18% based on a viral surrogate.^37^ These simulated nose contacts were assumed to be with the mucosal membrane as opposed to other parts of the nose, such as the bridge of the nose, that would not result in a dose. The fractional surface area of contact ($S_{m}$) was assumed to be equal to one fingertip. To estimate this surface area, the minimum and maximum front partial fingertip fractional surface areas were divided by 5 to inform the minimum and maximum values of a uniform distribution.^24^ The surface area of a hand ($A_{h}$) was randomly sampled from a uniform distribution with a minimum of 445 cm^2^ and a maximum of 535 cm^2^ ^19^ and is informed by the values from the Environmental Protection Agency, USA’s Exposure Factors Handbook.^38^ The expected inactivation of the virus during this contact assumed a single second contact, and the final $k_{h}$ value used in the care episode simulation was used. Δt represents the time between the doffing and touching the mucosa. A total of 10 000 parameter combinations are obtained for each care type scenario in a Monte Carlo framework.

### 2.9 Dose–response

Due to the lack of dose‐response curve data for SARS‐CoV‐2, an exact beta‐Poisson dose–response curve^39^ was fitted to pooled data for SARS‐CoV‐1 and HCoV 229E, assuming the infectivity of SARS‐CoV‐2 lies between the infectivity for these two organisms. In Equation (6), _1_ *F*_1_ (α,α+β,‐d) is the “Kummer confluent hypergeometric function” and P(d) is the probability of infection risk given dose^39^:

(6) $$P\left(d\right)=1{‐}_{1}F_{1}\left(\alpha ,\alpha +\beta ,‐d\right)$$

**FIGURE 1:**
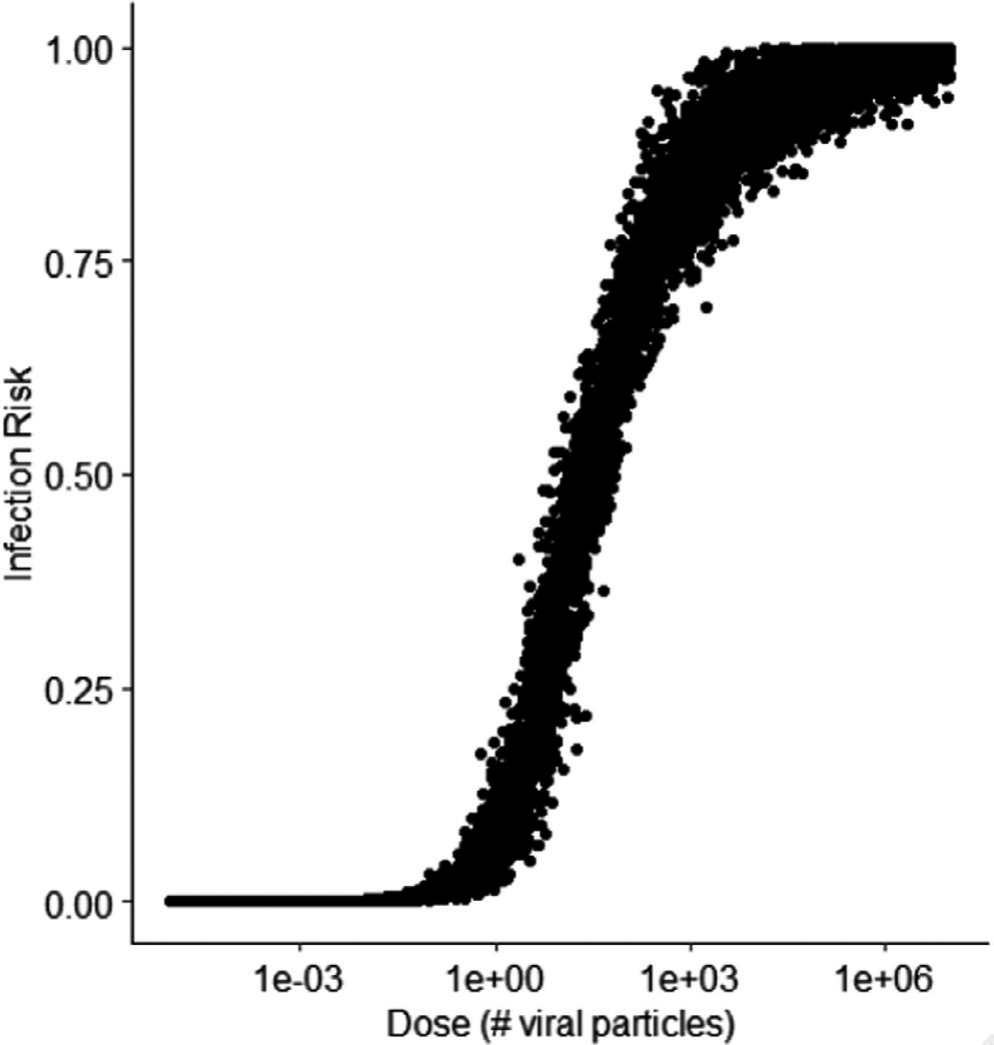
Dose‐response risk curve for averaged SARS CoV‐1 and Coronavirus 229E response

Ten‐thousand bootstrapped pairs of *α* and *β* were produced based on a maximum likelihood estimation fit. For each estimated dose, an *α* and *β* pair were randomly sampled, and an infection risk was estimated with Equation (6). The infectious dose for 50% of infections to occur was between 5 and 100 infectious viral particles with a mean of 30; the dose–response curve can be seen in Figure 1. We use this dose‐response curve within the discussion section as a comparator against the curve for HCoV229E also given in Ref. [39] which is considered a similar but more infectious virus.

### 2.10 Sensitivity analysis

Spearman correlation coefficients were used to quantify monotonic relationships between input variables and viral exposure. This method has been used in other QMRA studies to evaluate the relationship between model inputs and outputs.^22^, ^40^, ^41^

## 3 RESULTS

**FIGURE 2:**
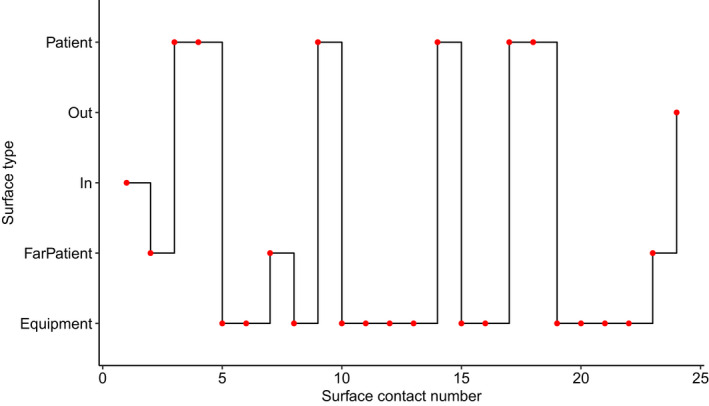
Stair plot of example HCW surface contacts during care, where “patient” is a hand‐to‐patient contact; “out” and “in” are exit and entrance into the patient room, respectively; “FarPatient” is a hand‐to‐far patient surface contact; and “Equipment” is a hand‐to‐equipment surface contact

Surface contact pattern predictions varied by care type. IV care resulted in the highest number of surface contacts (mean = 23, SD = 10) per episode, while observational care and doctors’ rounds had on average 14 (SD = 7) and 20 (SD = 6) contacts, respectively. A stair plot showing an example HCW surface contact pattern derived from the Markov chain prediction can be seen in Figure 2.

### 3.1 Estimated dose

**FIGURE 3:**
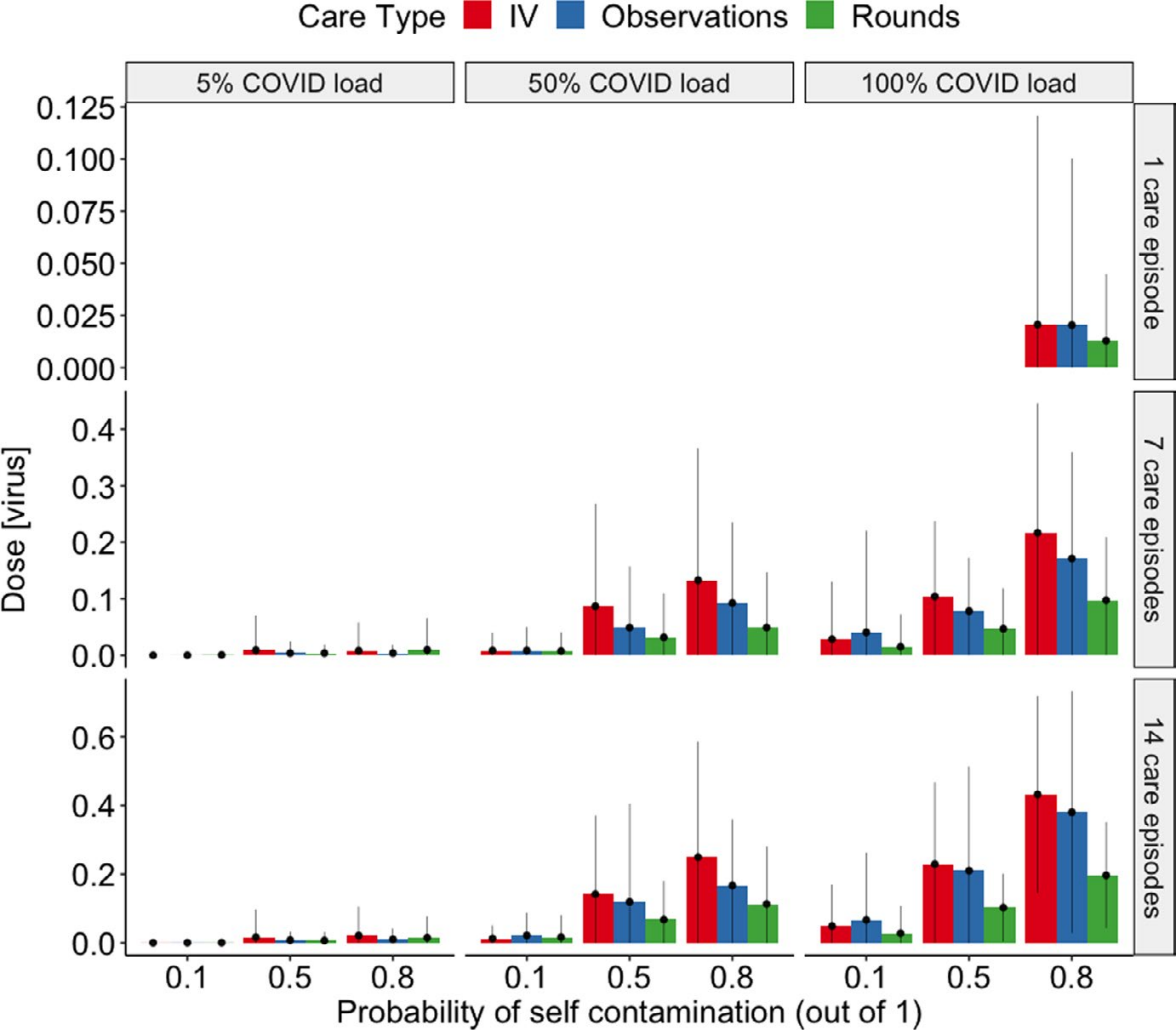
Bar chart showing dose per shift for IV, observations, and doctors’ rounds for different COVID patient loads. Error bars represent the standard deviation of the mean

Dose values in Table 2 and Figure 3 are given in a number of virus plaque‐forming units (PFU), where we also include all fractional values since these would correspond to multiple viruses for a higher surface load relating to different SARS CoV2 variants.

**TABLE 2:** PFU doses for each care type

Quantile	IV care	Observations	DRS' rounds
0%	0	0	0
25%	0	0	0
50%	0.00184	0.0021	0.00127
75%	0.0751	0.0651	0.0409
95%	0.506	0.421	0.234

Median PFU values for each care type were within the same order of magnitude (see Table 2), while maximum values for IV drip were 47% higher than for observations and 68% than for Drs’ rounds which can be explained by the number of surface contacts (IV‐drip care: 23 ± 10, doctors’ rounds: 14 ± 7 and observational care: 20 ± 6). Doubling patient load, regardless of COVID‐19 prevalence, probability of self‐inoculation or care type, caused median viral dose to increase by an order of magnitude from 0.0004PFUs to 0.0069PFUs (95%CI = 0 to 0.501PFU). Figure 3 shows a bar chart with standard deviations for care type, COVID‐19 prevalence on the ward, and chance of self‐contamination.

A linear regression of dose on all predictor variables conducted in R (version 4.0.1) shows that dose does not track linearly with COVID‐19 prevalence (*p *< 0.001), where the median dose received during 100% COVID‐19 prevalence was an order of magnitude higher than at 50% (0.008 PFU vs. 0.031PFU) and 0PFU aftercare with a ward of 5% COVID‐19 patients.

Spearman correlation coefficients for input parameters vs. viral dose received are given in Table 2. In terms of most important factors determining exposure, surface cleanliness was found to be the single most important, with hand‐to‐mouth/eyes/nose transfer efficiency only half as important (correlation coefficient *ρ* = 0.29 vs. *ρ* = 0.12, respectively) (see Table 3). Surface concentration relates to cleaning frequency; hence, the control case was run for half the surface bioburden.

**TABLE 3:** Spearman correlation coefficients of input parameters with infection risk

Parameter	Spearman correlation coefficient
Concentration on surfaces (viral particles/cm^2^)	0.27
Transfer efficiency to mouth, eyes, or nose^b^	0.08
Transfer efficiency surface to hand	0.03
Transfer efficiency hand to surface	0.01
Inactivation constant for surfaces	−0.02
Fraction of total hand surface area in contact	−0.02
Fraction of RNA relating to infectious particles^a^	0.04
Fraction of total hand surface area used in hand‐to‐face contact^b^	0.03
Total hand surface area^b^	0.02
Inactivation constant for hands	0.02

^a^ The spearman correlation coefficient represents instances where contacts with surfaces that had non‐zero concentrations were made.

^b^ The spearman correlation coefficient represents instances in which these parameters were used in a simulation where a contaminated hand‐to‐face contact was made after doffing.

## 4 DISCUSSION

### 4.1 Key findings and generalizability

The model developed in this study indicates that the exposure from mistakes after doffing PPE is likely to be low for a single shift, even for nurses on 100% patient COVID‐19 positive wards. Exposure doses vary by care type as greater frequencies of surface contacts directly impact viral loading on gloves and subsequent self‐contamination exposures. The dose increases further if error rates in doffing are high and a high proportion of patients are COVID‐19 positive (Figure 3), which highlights the importance of optimal hand hygiene, especially after PPE doffing.

Surface cleanliness was the most important factor in predicting dose regardless of doffing mistake likelihood, highlighting the relevance of frequency of cleaning regimes for managing risk. Halving the surface viral concentration decreased the exposure twofold. Studies have shown that microorganisms can be readily transferred between touch sites in a healthcare environment by routine activities.^42^ Dispersion of respiratory droplets and aerosols may contaminate less frequently touched surfaces as well, particularly where the patient is undergoing treatment that generates aerosols such as continuous positive airway (CPAP) ventilation. Sampling in COVID‐19 wards suggests aerosol deposition is a contributor to surface contamination, as one study has reported deposition at a distance of 3m from the patient.^11^ Previous experimental work aerosolizing bacteria in an air‐conditioned hospital room test chamber showed that surfaces well outside the patient zone can become contaminated with infectious material.^43^, ^44^ Since the observational study underlying the Markov chains reveals that at least 10% of staff contacts impact on such surfaces (excluding door handles), then the current lists of high‐touch surfaces^45^ that had historically been prioritized for cleaning, may need to be revised.

**FIGURE 4:**
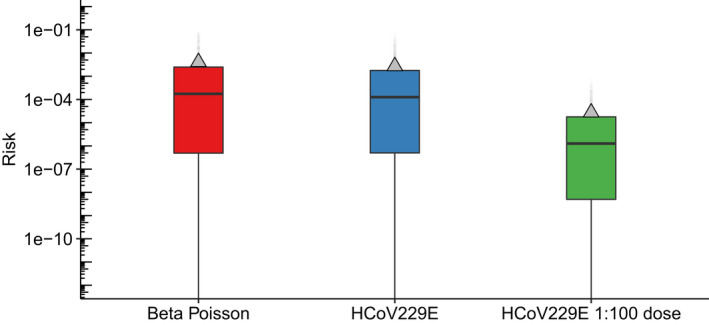
Boxplot showing Infection risk (i.e., individual probability of infection for each predicted dose), using the Beta‐Poisson and HCoV‐229E exponential dose‐response curve.^46^ Triangles represent the mean values

A dose‐response curve for SARS‐CoV‐2 is not yet available; furthermore, the contribution of each dose (i.e., upper respiratory vs. lower respiratory route) to individual infection risk may still be unclear even if and when it is obtained.^46^ Consequently, we have analyzed the results from the contact model based on relative exposures and qualitative trends to try and understand the effect of key parameters and mitigation strategies. In Figure 4, we plotted the risk [0–1] for each of the doses that the nurses received. We compare the prediction between the Beta Poisson dose‐response curve presented above against that for HCoV229E. We also follow the approach from Lei et al. and assume that the dose required for infection from the upper respiratory tract relating to a mucosal contact is 100 times higher than a dose reaching the lower respiratory tract.

In general, the mean risk is higher than the upper quartile alluding to the hypothesis that a few nurses may become infected which relates to opportunistic or rare events under these circumstances. Using a Bernoulli distribution with either a 1 or a 0 response, representing an infection or not from each one of the predicted exposure doses and corresponding individual infection risk probabilities, we can predict the number of nurses infected per 100 nurses.

From the individual risks predicted using the Beta‐Poisson curve and under a baseline assumption of 5% COVID‐19 positive patients, 14 care episodes, 10% chance of self‐inoculation, we see that 1 nurse is likely to become infected with another 1 possible based on the mean and standard deviations obtained from 100 Bernoulli simulation runs. Under the worst‐case scenario which could be roughly interpreted as an out‐of‐control epidemic in the community (100% COVID‐19 patients, 14 care episodes, 80% chance of self‐inoculation), this mean increases to 4 per 100 with a standard deviation of 4 infections.

The results in Figure 4 are illustrative to demonstrate the potential variability in infection risk that could result from exposures during a shift, but it is important to recognize that analysis of infection risk also needs to be interpreted in the context of the current status of the pandemic within a particular country or region. The emergence of more transmissible variants is already changing the exposure‐risk relationships, and it is likely that dose‐response will be specific to a particular variant. The risk of infection will also be substantially impacted by the vaccination status within a community. At the time of writing, 45 million people had received the first vaccine dose and 34 million, the second dose in the UK, which will substantially reduce the likelihood of infection further than those illustrated here.

Regardless of the number of COVID‐19‐positive patients on a ward, notable decreases in predicted infection risk were associated with less self‐contamination during doffing. For example, for scenarios involving all COVID‐19 patients, the mean infection risk for 10% probability of self‐contamination while doffing was 0.4%, while the mean infection risk for an 80% probability of self‐contamination while doffing was more than a 420% increase at 2.1%. This emphasizes the importance of adequate training for PPE use. Less risk of self‐contamination will decrease the transmission risks, potentially through sanitizing gloves with alcohol gel before doffing. PPE can be an effective strategy for mitigating exposure if proper doffing techniques are used. In addition to training, improvements in PPE design that enhance safety and expediency of doffing may lower self‐contamination rates and, therefore, improve PPE as a mitigation strategy.^47^ For example, fasteners or ties on gowns/masks were identified as “doffing barriers” because it was unclear whether these were to be untied and there were difficulties in reaching these ties. Self‐contamination due to gowns and masks was not specifically addressed in this model. It is possible that self‐contamination during doffing of items other than gloves could increase the potential risks due to incorrect doffing. Shortages of PPE have changed the normal practice where PPE is worn on a sessional basis rather than renewed for each patient. This means less doffing and potentially less auto‐contamination but may increase the risk of virus transfer within the unit.

In addition to the importance of safe and proper doffing, the results from this computational study also emphasize the importance of surface decontamination and environmental monitoring strategies. The concentration of virus on surfaces was the most influential parameter on the dose, which is consistent with other surface exposure studies.^22^ While SARS‐CoV‐2 RNA has been detected on surfaces, one limitation to a molecular approach is the lack of information regarding infectivity. In a recent study by Zhou et al. (2020), no surface samples demonstrated infectivity. However, it was noted that the concentrations of SARS‐CoV‐2 on the surfaces were below the current detection limits for culture methodologies.^32^ While there are known relationships between cycle threshold values and probabilities of detecting a viable virus in a sample,^48^, ^49^ it is necessary to know what fraction of detected genome copies relate to viral particles for QMRAs. More data are needed to better understand how molecular concentrations, even concentrations below detection limits, relate to infectivity and subsequent infection risk.

### 4.2 Model uncertainties

The model in this study only evaluates a surface transmission route while in reality, risks posed to healthcare workers are due to combined exposure pathways: air, droplet, person‐to‐person, and surface transmission. As the model only evaluates surface transmission, these infection risks are likely to be an underestimate of the total risk incurred by the healthcare workers over an entire shift. In a study of healthcare workers in a facility in Wuhan, China, 1.1% (110/9684) were COVID‐19 positive.^53^ According to CDC, from February 12–April 9, 2020, 19% (9282/49 370) of COVID‐19 US cases for which healthcare professional status was available, were healthcare workers.^54^ However, it is not known how many shifts were associated with these infection rates. Additionally, we assumed that wards with non‐COVID‐19 patients did not have SARS‐CoV‐2 contamination on surfaces, due to lack of data on SARS‐CoV‐2 surface contamination beyond COVID‐19 wards or patient rooms. There is a potential for asymptomatically infected healthcare workers to contribute to environmental contamination, especially when considering the relatively long shedding durations for asymptomatic infections.^55^ Infected healthcare workers and environmental contamination could be considered in future extensions of this model.

The fact that the proportions of healthcare workers with COVID‐19 discussed above are much larger than the infection risks estimated suggest that other transmission routes could drive additional HCW cases. This would include more transmission through airborne routes, or HCW to HCW transmission by asymptomatic cases outside the COVID‐19 care environment.^56^ However, while there continues to be disagreement over the contribution of each route to overall risk, transmission routes influence each other, making them all significant in healthcare environments. For example, surfaces can become contaminated due to the deposition of aerosolized virus. Viruses can later be resuspended from surfaces, contributing to air contamination. Future work should extend current models with a multi‐exposure pathway approach. This will advance not only our understanding of SARS‐CoV‐2 transmission but the transmission of pathogens in built environments as a whole.

It should be noted that there is still a large variation in gowns and masks and that there is the possibility of double gloving; hence, potentially reducing the risk of self‐contamination and the type of material and the design will also to an extent, determine the contamination risk.

Finally, a dose–response curve informed by SARS‐CoV‐1 and HCoV‐229E data was utilized, due to the lack of SARS‐CoV‐2‐specific dose‐response data. Despite limitations related to the dose‐response, the conclusions from the estimated doses were consistent with the insights from infection risk estimates. Increases in the probability of contamination between care episodes related to increases in the dose and most notably, for scenarios in which more than 5% of patients had COVID‐19 (Figure 3).

## 5 CONCLUSION

We propose a model for predicting exposure to healthcare workers from self‐contamination during the doffing of personal protective equipment over a single shift. The model estimates the quantity of SARS‐CoV‐2 virus accretion on gloved hands for three types of non‐aerosol‐generating procedures: IV‐care, observations, and doctors’ rounds. Once doffing was in progress, staff self‐contaminated a fraction of the times based on patient‐load fatigue. Three COVID‐19 positive patient scenarios (5%, 50%, and 100% COVID‐19 patients) were investigated amounting to a total of 30 000 parameter combinations allowing us to conduct a “what‐if” parametric study and sensitivity analysis. Surface viral concentration was found to be more than twice as important as any other factor whereby highlighting the importance of time‐appropriate cleaning. Transfer efficiency from finger to the nose was of secondary importance, although hand hygiene following doffing is still highly recommended. While the exposure from this type of self‐contamination is low per healthcare worker shift, this highlights that the procedures, if carried out correctly, are generally safe. It is accepted that other routes of transmission will play a significant role in infection propagation.

## CONFLICTS OF INTEREST

None to declare.

## AUTHOR CONTRIBUTIONS

Conceptualization: King and Wilson: equal. Formal analysis: King and Wilson: equal. Data curation: King and Proctor: equal. Funding: Noakes, López‐García: equal. Methodology: King and Wilson equal. Visualization: King lead. Writing: King and Wilson lead. Writing review: All authors equally.

### PEER REVIEW

The peer review history for this article is available at https://publons.com/publon/10.1111/ina.12938.

## References

[1] Johns Hopkins University . COVID‐19 Dashboard by the Center for Systems Science and Engineering (CSSE) at Johns Hopkins University. 10.1016/S1473-3099(22)00434-0 PMC9432867 36057267

[2] Boone SA , Gerba CP . Significance of fomites in the spread of respiratory and enteric viral disease. Appl Environ Microbiol. 2007;73:1687‐1696. 17220247 10.1128/AEM.02051-06 PMC1828811

[3] Otter JA , Donskey C , Yezli S , Douthwaite S , Goldenberg SD , Weber DJ . Transmission of SARS and MERS coronaviruses and influenza virus in healthcare settings: the possible role of dry surface contamination. J Hosp Infect. 2016;92:235‐250. 26597631 10.1016/j.jhin.2015.08.027 PMC7114921

[4] Kraay ANM , Hayashi MAL , Hernandez‐Ceron N , et al. Fomite‐mediated transmission as a sufficient pathway: a comparative analysis across three viral pathogens. BMC Infect Dis. 2018;18:540. 30373527 10.1186/s12879-018-3425-x PMC6206643

[5] Santarpia JL , Rivera DN , Herrera V , et al. Transmission potential of SARS‐CoV‐2 in viral shedding observed at the University of Nebraska Medical Center. medRxiv. 2020.03.23.20039446.

[6] Ye G , Lin H , Chen L , et al. Environmental contamination of the SARS‐CoV‐2 in healthcare premises: an urgent call for protection for healthcare workers. medRxiv. 2020.

[7] van Doremalen N , Bushmaker T , Morris DH , et al. Aerosol and surface stability of SARS‐CoV‐2 as compared with SARS‐CoV‐1. N Engl J Med. 2020;382:1564‐1567. 32182409 10.1056/NEJMc2004973 PMC7121658

[8] Casanova LM , Erukunuakpor K , Kraft CS , et al. Assessing viral transfer during doffing of Ebola‐level personal protective equipment in a biocontainment unit. Clin Infect Dis. 2018;66:945‐949. 29471475 10.1093/cid/cix956 PMC6927896

[9] Tomas ME , Kundrapu S , Thota P , et al. Contamination of health care personnel during removal of personal protective equipment. JAMA Intern Med. 2015;175:1904‐1910. 26457544 10.1001/jamainternmed.2015.4535

[10] Ong SWX , Tan YK , Chia PY , et al. Air, surface environmental, and personal protective equipment contamination by Severe Acute Respiratory Syndrome Coronavirus 2 (SARS‐CoV‐2) from a symptomatic patient. JAMA. 2020;323:1610. 32129805 10.1001/jama.2020.3227 PMC7057172

[11] Liu Y , Ning Z , Chen YU , et al. Aerodynamic analysis of SARS‐CoV‐2 in two Wuhan hospitals. Nature. 2020;582(7813):557‐560. 32340022 10.1038/s41586-020-2271-3

[12] Bullard J , Dust K , Funk D , et al. Predicting Infectious Severe Acute Respiratory Syndrome Coronavirus 2 From Diagnostic Samples. Clinical Infectious Diseases. 2020;71(10):2663–2666. doi:10.1093/cid/ciaa638 32442256 PMC7314198

[13] WHO . Quantitative Microbial Risk Assessment: Application for Water Safety Management. WHO Press; 2016:187.

[14] King MF , Noakes CJ , Sleigh PA . Modeling environmental contamination in hospital single‐ and four‐bed rooms. Indoor Air. 2015;25:694‐707. 25614923 10.1111/ina.12186 PMC4964916

[15] King MM‐F , López‐García M , Atedoghu KP , et al. Bacterial transfer to fingertips during sequential surface contacts with and without gloves. Indoor Air. 2020;30:993‐1004. 32329918 10.1111/ina.12682

[16] Wilson AM , Reynolds KA , Sexton JD , Canales RA . Modeling surface disinfection needs to meet microbial risk reduction targets. Appl Environ Microbiol. 2018;84:1‐9. 10.1128/AEM.00709-18 PMC6121971 29980557

[17] Wilson AM , Reynolds KA , Jaykus LA , Escudero‐Abarca B , Gerba CP . Comparison of estimated norovirus infection risk reductions for a single fomite contact scenario with residual and nonresidual hand sanitizers. Am J Infect Control. 2020;48(5):538‐544. 31676157 10.1016/j.ajic.2019.09.010

[18] Wilson AM , Reynolds KA , Canales RA . Estimating the effect of hand hygiene compliance and surface cleaning timing on infection risk reductions with a mathematical modeling approach. Am J Infect Control. 2019;47:1453‐1459. 31331717 10.1016/j.ajic.2019.05.023

[19] Beamer PI , Plotkin KR , Gerba CP , Sifuentes LY , Koenig DW , Reynolds KA . Modeling of human viruses on hands and risk of infection in an office workplace using micro‐activity data. J Occup Environ Hyg. 2015;12:266‐275. 25436665 10.1080/15459624.2014.974808 PMC4455933

[20] King M‐F , Wilson AM , López‐García M , et al. Why is mock care not a good proxy for predicting hand contamination during patient care?. Journal of Hospital Infection. 2021;109:44–51. doi:10.1016/j.jhin.2020.11.016 33271214

[21] Jinadatha C , Villamaria FC , Coppin JD , et al. Interaction of healthcare worker hands and portable medical equipment: a sequence analysis to show potential transmission opportunities. BMC Infect Dis. 2017;17:1‐10. 29281998 10.1186/s12879-017-2895-6 PMC5745722

[22] Julian TR , Canales RA , Leckie JO , Boehm AB . A model of exposure to rotavirus from nondietary ingestion iterated by simulated intermittent contacts. Risk Anal. 2009;29:617‐632. 19187484 10.1111/j.1539-6924.2008.01193.x

[23] King MF , Noakes CJ , Sleigh PA , Bale S , Waters L . Relationship between healthcare worker surface contacts, care type and hand hygiene: an observational study in a single‐bed hospital ward. J Hosp Infect. 2016;94:48‐51. 27392977 10.1016/j.jhin.2016.05.003

[24] Sizun J , Yu MWN , Talbot PJ . Survival of human coronaviruses 229E and OC43 in suspension and after drying on surfaces: a possible source of hospital‐acquired infections. J Hosp Infect. 2000;46:55‐60. 11023724 10.1053/jhin.2000.0795 PMC7134510

[25] Harbourt DE , Haddow AD , Piper AE , et al. Modeling the stability of severe acute respiratory syndrome coronavirus 2 (SARS‐CoV‐ 2) on skin, currency, and clothing. medRxiv. 2020. 10.1371/journal.pntd.0008831 PMC7676723 33166294

[26] AuYeung W , Canales RA , Leckie JO . The fraction of total hand surface area involved in young children’s outdoor hand‐to‐object contacts. Environ Res. 2008;108:294‐299. 18760778 10.1016/j.envres.2008.07.010

[27] Biryukov J , Boydston JA , Dunning RA , et al. Increasing temperature and relative humidity accelerates inactivation of SARS‐CoV‐2 on surfaces. mSphere. 2020;5(4):1‐9. 10.1128/mSphere.00441-20 PMC7333574 32611701

[28] Guo Z‐D , Wang Z‐Y , Zhang S‐F , et al. Aerosol and surface distribution of severe acute respiratory syndrome coronavirus 2 in hospital wards, Wuhan, China, 2020. Emerg Infect Dis. 2020;26. 10.3201/eid2607.200885 PMC7323510 32275497

[29] Pancic F , Carpentier DC , Came PE . Role of infectious secretions in the transmission of rhinovirus. J Clin Microbiol. 1980;12:567‐571. 6252242 10.1128/jcm.12.4.567-571.1980 PMC273638

[30] Whiteley GS , Glasbey TO , Fahey PP . A suggested sampling algorithm for use with ATP testing in cleanliness measurement. Infect Dis Heal. 2016;21:169‐175.

[31] Public Health England . Detection and enumeration of bacteria in swabs and other environmental samples. Natl Infect Serv Food Water Environ Microbiol Stand Method 4. 2017.

[32] Margas E , Maguire E , Berland CR , Welander F , Holah JT . Assessment of the environmental microbiological cross contamination following hand drying with paper hand towels or an air blade dryer. J Appl Microbiol. 2013;115:572‐582. 23683001 10.1111/jam.12248

[33] Zhou J , Otter JA , Price JR , et al. Investigating SARS‐CoV‐2 surface and air contamination in an acute healthcare setting during the peak of the COVID‐19 pandemic in London. Clin Infect Dis. 2020;1‐24. 10.1093/cid/ciaa905 PMC7454437 32634826

[34] Girou E , Loyeau S , Legrand P , Oppein F , Brun‐Buisson C . Efficacy of handrubbing with alcohol based solution versus standard handwashing with antiseptic soap: randomised clinical trial. BMJ. 2002;325:362. 12183307 10.1136/bmj.325.7360.362 PMC117885

[35] Kampf G , Todt D , Pfaender S , Steinmann E . Persistence of coronaviruses on inanimate surfaces and its inactivation with biocidal agents. J Hosp Infect. 2020;104:246‐251. 32035997 10.1016/j.jhin.2020.01.022 PMC7132493

[36] Van Abel N , Schoen ME , Kissel JC , Meschke JS . Comparison of Risk Predicted by Multiple Norovirus Dose‐Response Models and Implications for Quantitative Microbial Risk Assessment. Risk Analysis. 2017;37(2):245–264. doi:10.1111/risa.12616 27285380

[37] Beamer PI , Luik CE , Canales RA , Leckie JO . Quantified outdoor micro‐activity data for children aged 7–12‐years old. J Expo Sci Environ Epidemiol. 2012;22:82‐92. 21989500 10.1038/jes.2011.34

[38] U.S. Environmental Protection Agency . Exposure Factors Handbook 2011 Edition (EPA/600/R‐09/052F). USEPA; 2011.

[39] Lopez GU , Gerba CP , Tamimi AH , Kitajima M , Maxwell SL , Rose JB . Transfer Efficiency of Bacteria and Viruses from Porous and Nonporous Fomites to Fingers under Different Relative Humidity Conditions. Applied and Environmental Microbiology. 2013;79(18):5728–5734. doi:10.1128/aem.01030-13 23851098 PMC3754157

[40] King M‐F , Camargo‐Valero M , Matamoros‐Veloza A , Sleigh P , Noakes C . An Effective Surrogate Tracer Technique for S. aureus Bioaerosols in a Mechanically Ventilated Hospital Room Replica Using Dilute Aqueous Lithium Chloride. Atmosphere. 2017;8(12):238. doi:10.3390/atmos8120238

[41] Huslage K , Rutala WA , Gergen MF , Sickbert‐Bennett EE , Weber DJ . Microbial Assessment of High‐, Medium‐, and Low‐Touch Hospital Room Surfaces. Infection Control & Hospital Epidemiology. 2013;34(2):211–212. doi:10.1086/669092 23295570

[42] Baloh J , Reisinger HS , Dukes K , et al. Healthcare workers’ strategies for doffing personal protective equipment. Clin Infect Dis. 2019;69:S192‐S198. 31517970 10.1093/cid/ciz613 PMC6743502

[43] Bullard J , Dust K , Funk D , et al. Predicting infectious SARS‐CoV‐2 from diagnostic samples. Clin Infect Dis. 2020;1‐18. 10.1093/cid/ciaa638 PMC7314198 32442256

[44] La Scola B , Le Bideau M , Andreani J , et al. Viral RNA load as determined by cell culture as a management tool for discharge of SARS‐CoV‐2 patients from infectious disease wards. Eur J Clin Microbiol Infect Dis. 2020;39:1059‐1061. 32342252 10.1007/s10096-020-03913-9 PMC7185831

[45] Lai X , Wang M , Qin C , et al. Coronavirus Disease 2019 (COVID‐2019) infection among health care workers and implications for prevention measures in a Tertiary Hospital in Wuhan. China. JAMA Netw Open. 2020;3:e209666. 32437575 10.1001/jamanetworkopen.2020.9666 PMC7243089

[46] Watanabe T , Bartrand TA , Weir MH , Omura T , Haas CN . Development of a dose‐response model for SARS coronavirus. Risk Anal. 2010;30:1129‐1138. 20497390 10.1111/j.1539-6924.2010.01427.x PMC7169223

[47] Centers for Disease Control and Prevention . Coronavirus Disease 2019 (COVID‐19): Cases in the U.S. 2020.

[48] Long Q , Tang X , Shi Q , et al. Clinical and immunological assessment of asymptomatic SARS‐CoV‐2 infections. Nat Med. 2020;26(8):1200‐1204. 32555424 10.1038/s41591-020-0965-6

[49] Sikkema RS , Pas SD , Nieuwenhuijse DF , et al. COVID‐19 in health‐care workers in three hospitals in the south of the Netherlands: a cross‐sectional study. Lancet Infect Dis. 2020;3099:1‐8. 10.1016/S1473-3099(20)30527-2 PMC7332281 32622380

[50] Rusin P , Maxwell S , Gerba C . Comparative surface‐to‐hand and fingertip‐to‐mouth transfer efficiency of gram‐positive bacteria, gram‐negative bacteria, and phage. J Appl Microbiol. 2002;93:585‐592. 12234341 10.1046/j.1365-2672.2002.01734.x

[51] Kasloff SB , Leung A , Strong JE , Funk D , Cutts T . Stability of SARS‐CoV‐2 on critical personal protective equipment. Scientific Reports. 2021;11(1). doi:10.1038/s41598-020-80098-3 PMC7806900 33441775

[52] Xie G , Roiko A , Stratton H , Lemckert C , Dunn PK , Mengersen K . Guidelines for use of the approximate beta‐Poisson dose–response model. Risk Anal. 2017;37:1388‐1402. 27704592 10.1111/risa.12682

[53] Canales RA , Wilson AM , Sinclair RG , et al. Microbial study of household hygiene conditions and associated Listeria monocytogenes infection risks for Peruvian women. Trop Med Int Heal. 2019;24:899‐921. 10.1111/tmi.13246 31066175

[54] Canales RA , Reynolds KA , Wilson AM , et al. Modeling the role of fomites in a norovirus outbreak. J Occup Environ Hyg. 2019;16:16‐26. 30274562 10.1080/15459624.2018.1531131

[55] Rawlinson S , Ciric L , Cloutman‐Green E . COVID‐19 pandemic – let’s not forget surfaces. J Hosp Infect. 2020;105(4):790‐791. 32445775 10.1016/j.jhin.2020.05.022 PMC7238988

[56] King M‐F , Noakes CJ , Sleigh PA , Camargo‐Valero MA . Bioaerosol deposition in single and two‐bed hospital rooms: a numerical and experimental study. Build Environ. 2013;59:436‐447.

